# A Visual Telerehabilitation Program in Virtual Reality for Age-Related Macular Degeneration: Randomized Feasibility and Proof-of-Concept Trial

**DOI:** 10.2196/87596

**Published:** 2026-08-03

**Authors:** Yulia Pyatova, Bill Zhang, Mariana Misawa, Samuel N Markowitz, Atri Sen, Lora Appel, Kyle Cheung, Pi Nasir, Danielle Tchao, Eduardo Garcia-Giler, Monica Daibert-Nido, Michael Reber

**Affiliations:** 1 University Health Network, Toronto Western Hospital, Toronto, ON, Canada; 2 University Health Network, Donald K. Johnson Eye Institute, 60 Leonard Avenue, Toronto, ON, M5T0S8, Canada, 1 416-634-7937; 3 School of Health Policy and Management, Faculty of Health, York University, Toronto, ON, Canada; 4 University Health Network, OpenLab, Toronto, ON, Canada

**Keywords:** macular degeneration, low vision, visual rehabilitation, biofeedback training, virtual reality

## Abstract

**Background:**

Age-related macular degeneration (AMD) causes progressive central vision loss in older adults. Low-vision rehabilitation can improve functional vision by training the use of a preferred retinal locus, commonly through clinic-based biofeedback training (BFT). However, repeated supervised rehabilitation is burdensome, and functional gains may be difficult to sustain without home practice. Stand-alone virtual reality (VR) may enable home-based, remotely monitored visual stimulation, but feasibility, safety, and usability in older adults with AMD remain insufficiently characterized.

**Objective:**

This study aimed to evaluate the feasibility and safety of adding home-based VR 3D single-object tracking (3D-SOT-VR) to conventional BFT in older adults with dry AMD in a parallel, randomized, single-blind (to assessors), controlled, formative trial and to generate exploratory functional hypotheses for a future trial.

**Methods:**

Adults with dry AMD were recruited at the Low Vision Clinic, Toronto Western Hospital, University Health Network, Toronto, Ontario, Canada, from September 2021 to October 2023. Participants were randomized to BFT once weekly for 4 weeks (BFT group) or BFT plus home-based 3D-SOT-VR (BFT-VR group) every other day for 4 weeks. Experimental intervention consisted of tracking a single object among distractors moving at different speeds in a 3D virtual space in a VR headset. Primary feasibility and safety outcomes included recruitment, adoption, adherence, compliance, intervention completion, remote data transfer, usability, and VR-induced symptoms and effects. Secondary outcomes included visual acuity, contrast sensitivity, fixation stability, retinal sensitivity, reading speed, and low-vision quality of life. Exploratory outcomes assessed performance at 3D-SOT-VR and usage. Analyses were descriptive and exploratory, with CIs and denominators reported to reflect limited precision and missingness.

**Results:**

Fourteen individuals were randomized (BFT, n=6; BFT-VR, n=8), below the planned sample size of 32. Recruitment was not achieved because of COVID-19–related interruptions and reduced onsite access. Eleven individuals were analyzed for the primary outcome (BFT n=6, BFT-VR n=5). Intervention completion was 100% in the BFT arm and 75% in the BFT-VR arm, below the prespecified BFT-VR threshold. Among participants who used VR, adherence to scheduled home sessions was acceptable, completed VR-session files were transmitted without loss, and no participant met the predefined cybersickness stopping rule. One BFT-VR participant discontinued because headset weight caused neck fatigue. Group-level visual outcomes did not provide significant effectiveness. Reading speed showed a clinically meaningful individual-level improvement in the BFT-VR arm and correlated with VR-task performance. The findings were not clearly durable at follow-up.

**Conclusions:**

This pilot study provides formative evidence that clinic-based BFT combined with home-based, remotely monitored VR visual stimulation can be implemented safely in older adults with dry AMD, while identifying major contextual feasibility barriers. The intervention is innovative because it extends low-vision rehabilitation into the home using a connected device and objective performance monitoring. Recruitment, retention, missing data handling, and sustainability of functional gains must be addressed before effectiveness testing.

## Introduction

### Background

Age-related macular degeneration (AMD) is the main cause of irreversible vision loss in developed countries, responsible for the loss of central, or macular, vision, substantially affecting reading, face recognition, mobility, independence, and quality of life [1,2]. According to BrightFocus Foundation, the number of people living with macular degeneration is expected to reach 196 million worldwide in 2020 and to increase to 288 million by 2040 [3]. There are no treatments available to restore vision and cure the disease; therefore, low-vision rehabilitation remains a central component of supportive care. Contemporary rehabilitation aims not only to prescribe magnification or optical aids, but also to improve the functional use of residual peripheral retina and to reduce the activity limitations caused by central scotoma [4,5]. A core concept in AMD rehabilitation is the preferred retinal locus (PRL), an eccentric retinal area that patients use to substitute for foveal fixation after macular damage [4]. Following the degeneration of the macula and the loss of central vision, patients naturally relocate the fixation point for high visual acuity (VA) peripherally, in an eccentric and healthy part of the retina, to compensate for visual impairments [6]. Relocation to the PRL occurs over time [4,7] but, in more than 25% of cases, the naturally developed PRL lands on a suboptimal retinal area and becomes inefficient, contributing to impaired reading and daily visual tasks [4,6,7]. Modern standard low-vision care for patients with AMD includes biofeedback training (BFT) to relocate the PRL to a healthy retinal patch identified by the health care provider and to acquire better fixation skills [4,7-9]. BFT has been shown to improve distance and near vision, reading speed, contrast sensitivity, retinal sensitivity, and therefore quality of life [4,7,10-12]. However, in clinical practice, it is observed that the patients do not always use the PRL as trained for activities of daily living, losing the benefits of BFT over time, while also highlighting uncertainties regarding optimal training dose, treatment interval, durability, and patient selection [9,13]. The use of the new PRL requires an adjustment of the oculomotor control [4,6,7] and a brain adaptation [14]. In patients with AMD, not only is fixation stability impaired at the PRL [6], negatively affecting VA [15] and making daily tasks such as reading and grooming difficult, but the increased eccentricity of a visual target also extends reaction times and multiplies error rates, deteriorating performance in object detection and recognition tasks [1,2,16]. These observations provide a rationale for combining conventional BFT with repeated attentional and visuomotor training outside the clinic. However, standard BFT is typically delivered under professional supervision in weekly or biweekly sessions, creating access and treatment-burden constraints for older adults. Home-based digital rehabilitation could increase training opportunities, but feasibility must be established before clinical effectiveness can be inferred.

### Objective

A new and promising vision rehabilitation technique using binocular 3D single object tracking (3D-SOT) deployed in virtual reality (VR) headsets can provide additional training of the ocular system [17-19]. This innovative technique is particularly well suited for training because the central features of the 3D-SOT closely match attentionally demanding real-life situations [20,21]. Three-dimensional object tracking stimulation programs displayed on monitors or television screens have been shown to increase brain capacity for complex processes such as anticipation, eye-tracking, field of view, and even decision-making in healthy and pathological participants [22-24]. Preliminary observations in low-vision patients with homonymous hemianopsia indicated a beneficial effect on visual perception of an audiovisual 3D object tracking VR stimulation program using stand-alone VR headsets at home [17,18,25]. We hypothesize that the intervention combining BFT and audiovisual 3D-SOT-VR stimulation may have an additive effect in restitution of visual functions and better visual skills resulting in an improved quality of life.

## Methods

### Study Design

This study was a 4-week, open-label, reference-controlled, randomized, feasibility, and proof-of-concept trial assessing feasibility (recruitment rates, adoption, adherence and compliance, data collection and retrieval, and safety) and potential effectiveness of BFT+3D-SOT-VR (BFT-VR group) versus BFT (BFT group) on visual perception in individuals with AMD and is reported following the CONSORT eHealth statement for pilot and feasibility studies.

### Ethical Considerations

The study protocol has been approved by the Research Ethics Boards at the University Health Network, Toronto, Ontario, Canada (UHN REB# 20‐6143) and registered on ClinicalTrials.gov (NCT04685824; posted on December 28, 2020) following the principles outlined in the World Medical Association Declaration of Helsinki: Research involving human subjects. All participants provided written informed consent before study procedures. The original informed consent allows the secondary analysis without additional consent. Study data were deidentified and pseudonymized for analysis, stored on secure institutional systems, and transmitted through the predefined secure study data management pathway. Participants received US $36 per visit as compensation for commute and parking expenses. No identifiable participant images are included in the paper or multimedia appendices. The study was conducted at the Ophthalmology service, Toronto Western Hospital, University Health Network, Toronto, Ontario, Canada, between September 15, 2021, and October 6, 2023, with multiple interruptions and extensions due to the Ontario Emergency Management and Civil Protection Act related to the COVID-19 pandemic [26].

### Participants

The study population included adults with dry AMD from the Toronto Western Hospital pool of patients who were referred by their ophthalmologists. Participants were enrolled by the co-principal investigator ophthalmologist. Inclusion criteria were diagnosed with dry AMD, male and female, VA (best-corrected visual acuity)<0.9 logMAR (logarithm of the minimum angle of resolution), ability to follow the visual and auditory stimuli and training instructions, online auditory test positive (−5 dBHL [decibels hearing level] to 30 dBHL range), home Wi-Fi access. Exclusion criteria were ocular disease not related to AMD physiopathology, wet AMD, both eyes with media opacity that impairs microperimetry testing, inability to perform during testing and training, consumption of psychoactive drugs, three consecutive Virtual Reality-Induced Symptoms and Effects (VRISE) questionnaire scores <25 at inclusion [27], history of vertigo or dizziness, and prior visual rehabilitation interventions.

### Randomization and Intervention

**Figure 1.**
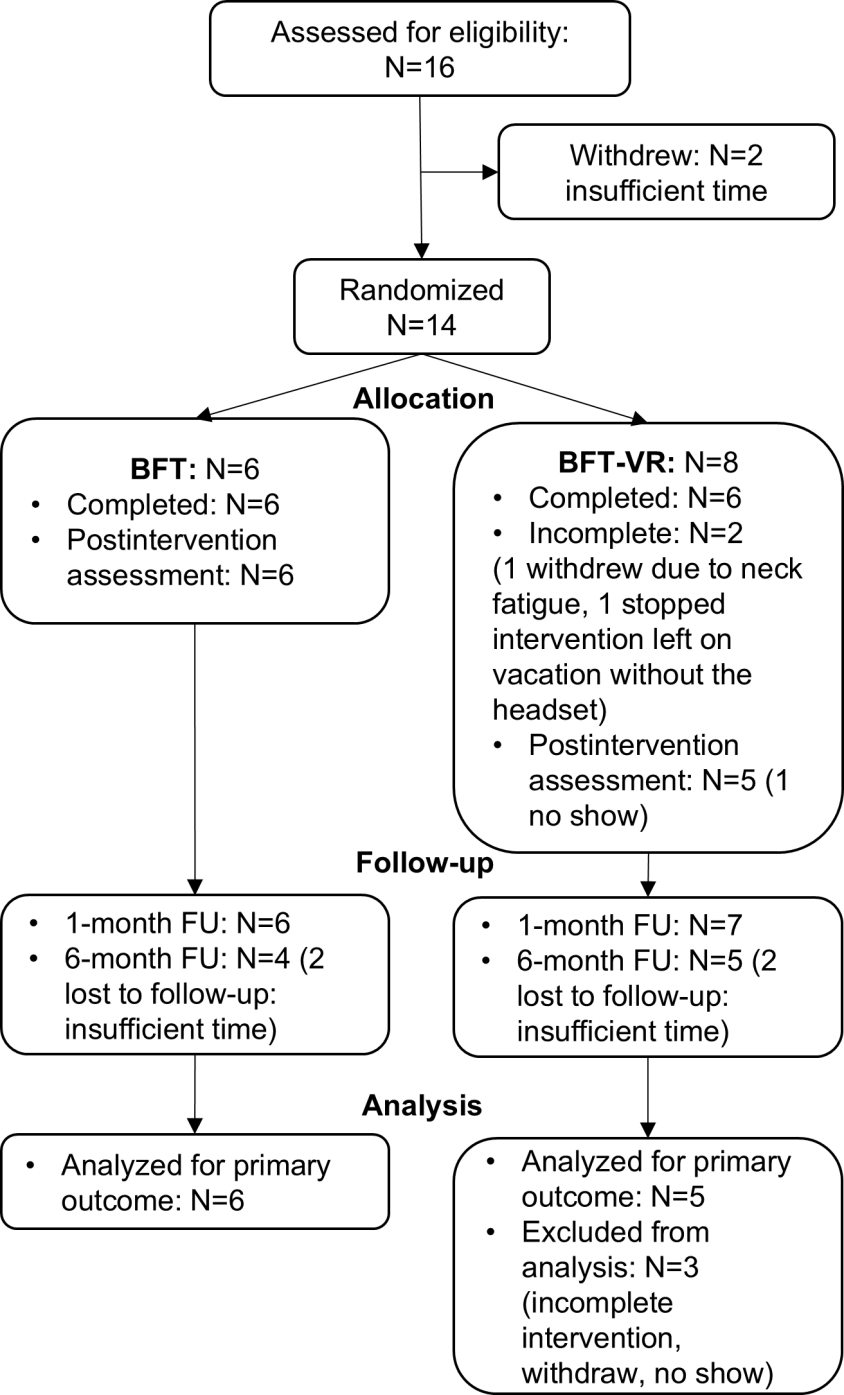
Study flowchart according to CONSORT (Consolidated Standards of Reporting Trials) 2025 flow diagram. BFT: biofeedback training; VR: virtual reality; FU: follow-up.

**Figure 2.**
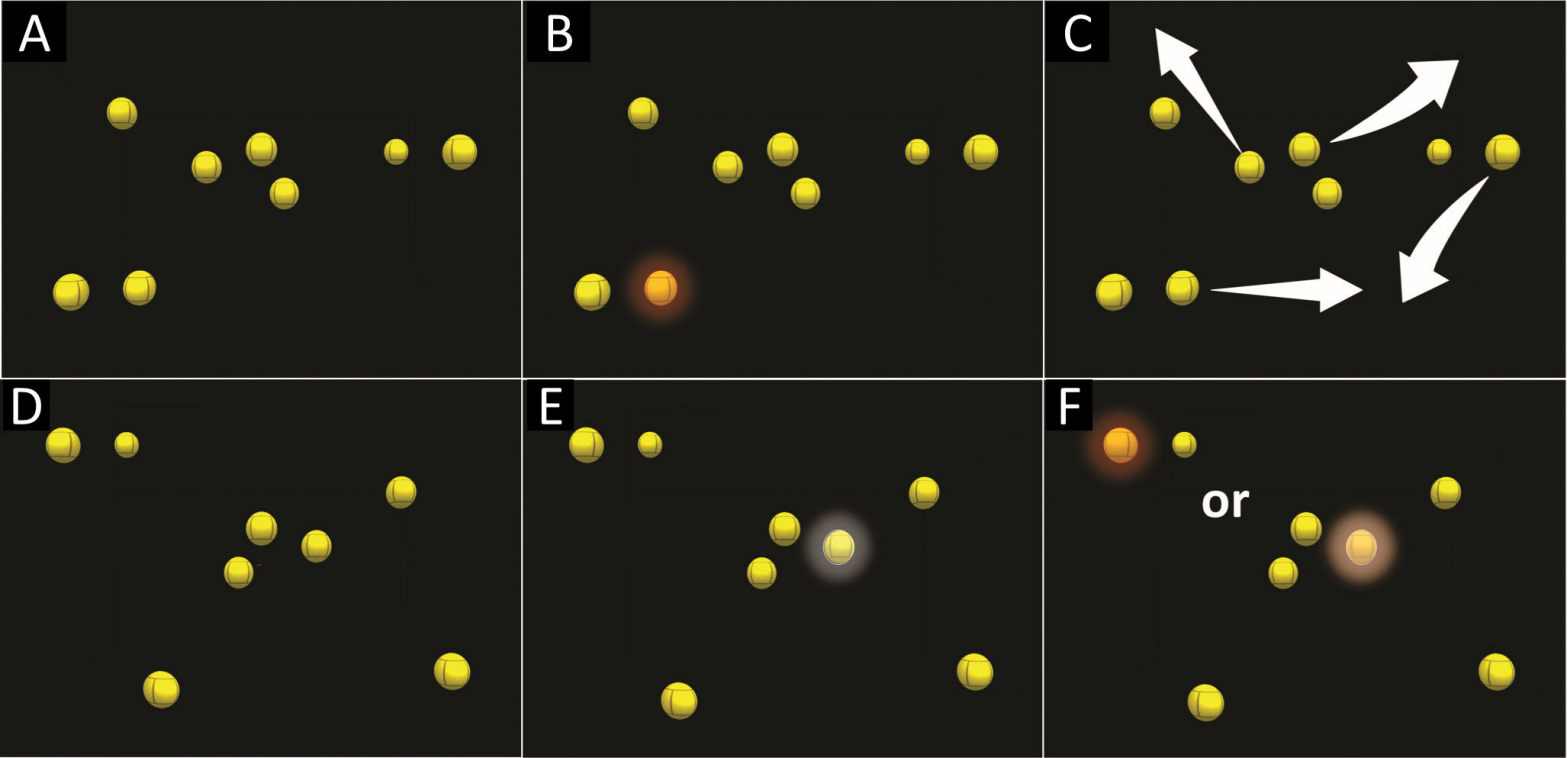
Dynamic audiovisual 3D single-object tracking (3D-SOT-VR) stimulation. (A) Eight yellow still spheres are present in a virtual cube. (B) One of these spheres turns red for 15 seconds (cued target) and returns yellow. (C) All spheres randomly move following linear paths across the visual field encompassing the blind field and bouncing off one another and off the walls of the virtual 3D cube when collisions occur. (D) After 30 seconds, spheres stopped moving. (E) The patient had to select the cued target using a hand-guided 32 laser pointer. (F) Correct selection is considered a positive hit.

Following guidelines for feasibility and pilot studies [28,29], the sample size was determined at 16 participants per arm. The participants were randomized 1:1 (permuted block, block size=2, 4, sealedenveloppe.com generated by the principal investigator) to receive audiovisual monocular (either right -oculus dexter, or left -oculus sinister, eye) BFT for 20 minutes once a week for 4 weeks at Toronto Western Hospital (reference-controlled group, BFT group) as described earlier [7], or audiovisual monocular BFT once a week for 4 weeks and binocular 3D-SOT-VR every other day at home for 4 weeks (interventional group, BFT-VR group), using the stand-alone and remotely controlled head-mounted display Meta Quest 2 (allowing for additional wearing of glasses) provided by our laboratory (Figure 1). Each session of stimulation is composed of three blocks of 15 trials of 20 seconds, equivalent to 3 × 5 minutes of continuous audiovisual stimulation corresponding to 16 sessions for a total time of 3 hours and 15 minutes of stimulation. The audiovisual stimulation task corresponds to the object-tracking paradigm, developed in the 1980s to study visual attention in humans [20,21,30]. The 3D-SOT is composed of eight high-contrast yellow spheres on a black background (luminosity=80 cd/m^2^, size=1°-3° of visual angle). After one of the spheres was cued (turning red for 5 s and switching back to yellow; Figure 2), the spheres moved for 20 seconds following random linear paths, bouncing off one another and off the walls of a virtual 3D cube when collisions occurred. The overall span of the movement of the spheres covers 80° and 70° horizontal and vertical visual angles, respectively, as measured using augmented reality and a geometric chart. The initial speed of the spheres was adjustable, from 3 to 240°/second, and determined individually for each participant at inclusion. A spatial sound (125 Hz, 0‐30 dBHL) is emitted by the cued target and spatially and temporally correlated to its movement. The maximum delay between sound and target is equivalent to the refreshing rate of the headsets, corresponding to 16.6 ms (temporal tolerance), below the 100 ms temporal disparity above which multisensory response enhancement decreases [31]. After 20 seconds, the movement stops and the participants are asked to select, using a virtual laser pointer, the cued sphere (the target) among the other spheres (mark-all procedure) [21]. If the selection is correct (corresponding to the cued target), a positive feedback sound is provided, and the speed of the spheres in the next trial is increased. If the selection is incorrect (corresponding to a distractor), a negative feedback sound is provided, and the speed of the spheres in the next trial is decreased. Speed is modified following an adaptive simple up-down staircase:±0.05 log [32,33]. Participants were allowed to use head and eye movements to track the moving target (overt tracking). During the home-based procedures, head or target position was recorded during the task by the Meta Quest 2 built-in head tracking system (sampling rate 60 Hz) downsampled to 10 Hz for analysis.

### Outcome Measures

Primary outcomes correspond to feasibility (recruitment rate, dropouts, adoption, adherence, and compliance) and safety (cybersickness). Secondary outcomes correspond to visual function and functional vision measured at the Ophthalmology Low Vision Clinic at the Toronto Western Hospital (University Health Network) following standard procedures in low-vision rehabilitation [5,34]. These visual assessments, blind to the assessor, include VA (best-corrected visual acuity) measured using the Early Treatment Diabetic Retinopathy Study and Colenbrander charts at 4 m; contrast sensitivity measured using the Functional Acuity Contrast Test. Fixation stability (bivariate contour ellipse area 63%) and retinal sensitivity were measured using the Macular Integrity Assessment microperimeter (CentreVue). Reading speed was measured at critical print size using the Minnesota Low Vision Reading test [35]. Quality of vision was measured using the Veterans Affairs–Low-Vision Visual Functioning Questionnaire [36].

Exploratory outcomes in the BFT-VR group corresponded to home-based VR-task performance, including target speed for correct selection, reaction time, head movement, and session time of day. These exploratory variables were collected remotely during the VR task and were used to evaluate operational feasibility and generate mechanistic hypotheses.

### Safety, Monitoring, Criteria for Removal, Early Stopping Rules, and Condition of Replacement

Monitoring of the at-home 3D-SOT-VR stimulation program occurred after each 5-minute block when the head-mounted display sent data relative to the participants’ adherence and compliance (number of tasks and number of blocks initiated and completed, duration of the blocks or sessions), performance (correct or incorrect selection ratio, reaction time, speed of the target, head movement, and time of day), and safety (VRISE – questionnaire scores before and after intervention) to our secured laboratory computer through Wi-Fi. Criteria for removal and stopping rules were 3 consecutive home-based VRISE scores postintervention ≤25 [27] or no 3D-SOT-VR stimulation reports for 5 consecutive days. There was no replacement for withdrawn participants.

### Data and Statistical Analysis

Data analysis of the primary outcomes was performed against predetermined feasibility and safety criteria. Data analysis on secondary outcomes was performed following both intention-to-treat (ITT) and per protocol (PP) methods, based on descriptive statistics including a measure of central tendency (mean) and dispersion (95% CI). Statistical comparisons between groups were performed using the Welch *t* test. Comparisons between pre- and postintervention data were performed using the Wilcoxon signed-rank test. Clinical meaningfulness was determined using the coefficient of repeatability (COR), specific to each test, to compare pre- and postintervention data [37]. The COR, also referred to as the smallest real difference, quantifies absolute reliability in the same unit as the assessment tool. The COR is directly related to the 95% limits of agreement proposed by Bland and Altman [38]. It corresponds to the value below which the absolute differences between two measurements would lie within 95% of probability. Therefore, measurement values strictly different from COR indicate an effect of the intervention [37,39]. Interaction effects were estimated using ANOVA.

### Missing Data and Missing Completely At Random Assessment

Missingness was summarized by study arm, visit, and outcome category. Missing data occurred mainly at the participant or visit level because of withdrawal, missed postintervention assessment, intervention discontinuation, and follow-up loss, rather than as isolated item-level missingness within completed visits. We assessed whether the observed missing data structure was compatible with a Missing Completely At Random (MCAR) mechanism, defined as missingness unrelated to both observed and unobserved data [40]. A formal Little MCAR test was considered [40], but the very small randomized sample, sparse missing data patterns, and number of repeated outcome variables made the test statistically underpowered and not reliably interpretable. Moreover, several missing observations had documented clinical or logistical explanations, making a strict MCAR assumption implausible. Multiple imputation was therefore considered but not implemented because the available sample was too small to support a stable imputation model across multiple outcomes and visits [41]. Analyses were consequently based on available observations, with denominators reported and secondary outcomes interpreted as exploratory.

## Results

### Feasibility and Safety

Feasibility criteria were defined based on previous studies using VR as an interventional tool [42,43] and CONSORT (Consolidated Standards of Reporting Trials) guidelines for feasibility trials [28]. Of 32 participants determined by power calculation, 18 were screened and 14 randomized in a 1:1 ratio to BFT (n=6) or BFT-VR (n=8; Figure 1 and Table 1).

**Table 1.** Participants’ demographics.

Group	Sample size, n	Age (years), mean (SD)	Sex ratio	Treatment history
BFT^a^	6	81.5 (5.5)	4F^b^, 2M^c^	Last anti-VEGF^d^ injection >12 months
BFT-VR^e^	8	83.2 (5.3)	6F, 2M	Last anti-VEGF injection >12 months

a BFT: biofeedback training.

b F: female.

c M: male.

d VEGF: vascular endothelial growth factor.

e BFT-VR: biofeedback training in immersive virtual reality.

Recruitment rate was below feasibility criteria (≥1.5 participants/week) for both arms, at 0.13 and 0.11 participants/week for BFT-VR and BFT, respectively (Tables 2 and 3). Overall, 6/6 participants (100%) completed the reference-controlled intervention in the BFT arm, and 6/8 participants (75%) completed the investigational intervention in the BFT-VR arm, not reaching feasibility criteria for intervention completion (≥80% of the participants; Table 2). In the BFT-VR arm, 6/8 participants performed 90.6% (87/96) of the home-based scheduled stimulation sessions (45 trials of 20 s), reaching feasibility for adherence and compliance criteria (≥80%). Remote safety and exploratory data collection during the sessions reached the feasibility threshold (≥90%) with 100% of the files collected (96/96) through Wi-Fi, indicating no loss of data. Safety related to the stimulation was assessed using the VRISE questionnaire score, which evaluates cybersickness [27]. VRISE scores post stimulation showed a total average of 33.8 (SD 0.4) out of 35 points, with no scores ≤25 indicating no adverse event (cybersickness symptoms scores criteria ≤3 consecutive VRISE scores ≤25) [27,44] (Table 2). Detailed analysis showed that, for a total of 96 sessions, among the five types of VRISE symptoms (nausea, disorientation, dizziness, fatigue, and instability), fatigue (Δ_post-pre_= −0.37, 95% CI −0.46 to −0.28) and dizziness (Δ_post-pre_= −0.25, 95% CI −0.16 to −0.34) were the most pronounced symptoms after stimulation, with scores of 6.31 (95% CI 6.11-6.51) and 6.60 (95% CI 6.45-6.75) respectively, although above the cybersickness threshold (Tables 2 and 3).

One drop-out was due to the intervention: the weight of the headset (Meta Quest 2) was reported by the participant as too heavy for the neck muscles, which became sore after the 15-minute session. Usability, measured using a modified version of the virtual reality neuroscience questionnaire [27], showed an overall score >40 above the minimum threshold indicating adequate quality of the software (Table 2). However, three items related to the use of the VR device itself but not to the stimulation, VR device adjustment with straps and pointer use and selection using the remote controller, did not reach the acceptable threshold (Tables 2 and 3), indicating potential interference with the completion of the home-based program.

**Table 2.** Feasibility scores.

	Intervention (BFT-VR)^a^^,^^b^ (n=8)	Reference-controlled (BFT; n=6)
Feasibility scores		
Recruitment rate, participants/wk^c^	0.13	0.11
Dropouts, n^d^	1	0
Adoption, (%, ratio)^e^	100% (8/8)	100% (6/6)
Adherence/compliance BFT (%, ratio)^e^	100% (28/28)	100% (24/24)
Adherence/compliance IVR (%, ratio)^e^	75% (96/128)	—^f^
Remote data collection through Wi-Fi (%, ratio)^g^	100% (96/96)	—

a BFT: biofeedback training.

b VR: virtual reality.

c criteria: ≥1.5.

d criteria: ≤3.

e criteria: ≥80%.

f Not applicable.

g criteria: ≥90%.

**Table 3.** Safety and usability scores.

Scores	Intervention (BFT-VR)^a^^,^^b^, average score (95% CI)
Feasibility scores^c^^,^^d^	
Nausea	−0.03 (−0.07 to 0.01) 6.93 (6.86 to 7.00)
Disorientation	−0.09 (0.00 to 0.18) 6.84 (6.75 to 6.93)
Dizziness	−0.25 (-0.34 to −0.16) 6.60 (6.45 to 6.75)
Fatigue	−0.37 (-0.46 to −0.28) 6.31 (6.11 to 6.51)
Instability	−0.02 (−0.07 to 0.03) 6.92 (6.86 to 6.98)
Total scores post-IVR^e^	−0.76 (−1.11 to −0.41) 33.8 (33.4 to 34.2)
Usability scores^f^^,^^g^	
Putting device on	5.0 (4.1 to 5.9)
Adjusting device	4.8 (4.0 to 5.6)
Quality of graphics	5.6 (4.9 to 6.3)
Quality of sound	5.4 (4.7 to 6.1)
Quality of VR technology	5.8 (5.2 to 6.4)
Use of the pointer	4.4 (3.1 to 5.70)
Selection using the pointer	4.6 (3.6 to 5.6)
Device recharging process	6.1 (5.6 to 6.6)
Average total scores post-IVR	42.9 (39.6 to 46.2)

a BFT: biofeedback training.

b VR: virtual reality.

c Average score post-IVR (95% CI).

d Criteria average score: >5, criteria total score: >25.

e IVR: immersive virtual reality.

f Average score (virtual reality neuroscience questionnaire).

g Criteria average score: >5, criteria total score: >40.

### Preliminary Effect of the Intervention

Effectiveness analyses followed the ITT principle, comprising all 16 randomized participants analyzed as allocated. Because 2 participants in the intervention arm discontinued the program after less than 80% of the sessions and one missed all posttreatment assessments, we prospectively defined a PP set that included participants who attended ≥80% of the sessions and contributed a valid primary-outcome measurement at 4 weeks. Performing both ITT and PP analyses is in line with ICH (International Conference on Harmonisation) E9 guidance (§5.2.3) on assessing the robustness of trial inferences [45], and with CONSORT 2010 recommendations [28] to report numbers analyzed for each set. The PP analysis is reported as a sensitivity check; the ITT analysis remains the primary basis for inference. Clinical relevance was determined using each test’s COR; changes exceeding COR are clinically significant [37].

At baseline, groups did not differ in near or distance VA (ITT [Welch *t* test, 2-tailed]: near VA: *t*_12.1_=0.75; *P*=.47; far VA: *t*_15.5_=0.64; *P*=.53; PP [Welch *t* test, 2-tailed]: near VA: *t*_12.9_=0.80; *P*=.44; far VA: *t*_16_=0.32; *P*=.75). Post training, no statistically significant group-level gains emerged in either metric, and between-arm (ITT and PP analyses; Tables 4 and 5). Nevertheless, individual COR-positive improvement was observed: 3 of 6 BFT participants improved by ≥0.2 logMAR in near vision and 1 improved for distance; in BFT-VR, 3 of 8 participants showed near-vision gains and 2 showed distance-vision gains. One- and six-month follow-up revealed stable mean acuity but individual fluctuation (Table S1 in Multimedia Appendix 1).

Fixation stability (bivariate contour ellipse area 63%) showed no difference between groups at baseline (ITT: Welsch *t* test: *t*_9.85_=1.63, *P*=.14; PP: Welsch *t* test: *t*_11.6_=1.69, *P*=.12). After four weeks, BFT recipients showed no mean change (ITT: Welsch *t* test: *t*_18.1_=1.37, *P*=.19; PP: Welsch *t* test: *t*_19.5_=0.86, *P*=.40; Tables 4 and 5) whereas the PP subset of BFT-VR displayed a COR-level worsening (Tables 4 and 5). Individually, two BFT participants worsened (COR>0.61 °^2^), and one was stable. In the BFT-VR group, three participants had normal fixation stability at baseline and one worsened after intervention (COR>0.61 °^2^); the others did not show significant changes. Measures at 1- and 6-month post intervention showed no changes (Table S1 in Multimedia Appendix 1).

**Table 4.** Measures of visual outcomes and statistical analysis comparing experimental (biofeedback training in immersive virtual reality) and reference-controlled (biofeedback training) interventions (intention-to-treat).

Function	Intervention (BFT-VR)^a^				Reference controlled (BFT)^b^			
	Baseline, mean (95% CI)	Postintervention, mean (95% CI)	Mean difference (95% CI)	Wilcoxon ranked test	Baseline, mean (95% CI)	Postintervention, mean (95% CI)	Mean difference (95% CI)	Wilcoxon ranked test
Visual function								
Visual acuity (near-LogMAR)^c^^,d^	0.43 (0.29 to 0.56)	0.33 (0.20 to 0.46)	−0.06 (0.17 to 0.05)	—^e^	0.59 (0.19 to 0.99)	0.50 (0.21 to 0.79)	−0.14 (0.30 to 0.03)	8.5^f^
Visual acuity (far-LogMAR)^g^	0.63 (0.51‐0.76)	0.65 (0.51 to 0.79)	−0.02 (−0.17 to 0.13)	7^h^	0.71 (0.4 to 1.01)	0.68 (0.40 to 0.95)	−0.04 (−0.21 to 0.14)	6^f^
Fixation stability (BCEA^i^ 63 % to °2)^j^	1.92 (1.22 to 2.62)	2.16 (0.89 to 3.44)	0.35 (−0.93 to 1.63)	11^h^	4.25 (1.34 to 7.17)	4.06 (1.73 to 6.39)	−0.57 (−3.34 to 2.20)	9^f^
Retinal sensitivity, dB^k^	12.3 (9.74 to 14.9)	13.1 (10.4 to 15.7)	0.03 (−0.73 to 0.80)	12^h^	10.1 (5.79 to 14.4)	9.48 (4.99 to 14.0)	−0.62 (−1.39 to 0.15)	7^f^
Functional vision								
Reading speed (WPM)^l^^,m^	99.6 (44.8 to 154.3)	105.0 (43.5 to 166.8)	13.2 (−10.8 to 37.3)	10^h^	82.5 (20.6 to 144.4)	76.1 (31.3 to 120.9)	−6.4 (−53.3 to 40.6)	7^f^
Quality of life								
LV-VFQ-48^n^^,o^								
Visual ability	1.22 (0.39 to 2.04)	1.56 (0.75 to 2.36)	0.29 (-0.12 to 0.69)	8^h^	1.51 (0.50 to 2.51)	1.92 (0.92 to 2.93)	0.42 (0.05 to 0.78)	2^p^
Reading	0.82 (-0.27 to 1.92)	1.70 (0.3 to 3.00)	0.92 (0.17 to 1.66)	2^q^	1.84 (0.51 to 3.17)	3.01 (0.76 to 5.26)	1.17 (0.06 to 2.28)	1^r^
Mobility	1.47 (0.77 to 2.18)	1.59 (0.97 to 2.21)	0.08 (-0.23 to 0.39)	13^h^	1.24 (0.28 to 2.21)	1.59 (0.57 to 2.62)	0.35 (−0.18 to 0.88)	4^f^
Visual information	1.46 (0.61 to 2.31)	1.54 (0.68 to 2.40)	0.01 (-0.61 to 0.64)	13^h^	1.67 (0.44 to 2.90)	2.02 (1.06 to 2.97)	0.35 (−0.12 to 0.83)	3^f^
Visual motor	1.09 (0.06 to 2.12)	1.24 (0.44 to 2.05)	0.13 (-0.41 to 0.68)	9^h^	1.40 (0.50 to 2.30)	1.64 (0.86 to 2.43)	0.24 (−0.32 to 0.80)	3^f^
COR 2.2^s^								
Total	6.02 (1.84 to 10.2)	7.70 (3.7 to 11.6)	1.68 (-0.46 to 3.81)	8^h^	7.66 (2.55 to 12.8)	10.2 (4.72 to 15.7)	2.54 (0.48 to 4.59)	2^p^

a BFT-VR: biofeedback training in immersive virtual reality.

b BFT: biofeedback training.

c LogMAR: logarithm of the minimum angle of resolution.

d COR=0.24.

e Not applicable.

f n=6; *P*>.2.

g COR=0.11.

h n=8; *P*>.2.

i BCEA: bivariate contour ellipse area.

j COR=0.61.

k COR=1.6.

l WPM: words per minute.

m COR=8.6.

n LV-VFQ-48: Low vision visual function questionnaire.

o COR=0.44.

p n=6.05<*P*<.1.

q n=8.02<*P*<.05.

r n=6.1<*P*<.2.

s COR: coefficient of repeatability.

**Table 5.** Measures of visual outcomes and statistical analysis comparing experimental (biofeedback training in immersive virtual reality) and reference-controlled (biofeedback training) interventions (per protocol).

Function	Intervention (BFT-VR)^a^				Reference controlled (BFT)^b^			
	Baseline, mean (95% CI)	Postintervention, mean (95% CI)	Mean difference (95% CI)	Wilcoxon ranked test	Baseline, mean (95% CI)	Postintervention, mean (95% CI)	Mean difference (95% CI)	Wilcoxon ranked test
Visual function								
Visual acuity (near-LogMAR)^c^^,^^d^	0.41 (0.25 to 0.57)	0.33 (0.16 to 0.50)	−0.07 (−0.23 to 0.08)	5^e^	0.59 (0.19 to 0.99)	0.50 (0.21 to 0.79)	−0.15 (−0.33 to 0.03)	8.5^f^
Visual acuity (far-LogMAR)^g^	0.66 (0.51 to 0.81)	0.59 (0.45 to 0.73)	−0.07 (−0.13 to −0.01)	1^h^	0.71 (0.42 to 1.01)	0.68 (0.40 to 0.95)	−0.04 (−0.21 to 0.14)	6^f^
Fixation stability (BCEA^i^ 63 % to °2)^j^	1.53 (0.65 to 2.41)	2.77 (0.98 to 4.56)	1.24 (-0.30 to 2.78)	2^h^	4.25 (1.34 to 7.17)	4.06 (1.73 to 6.39)	−0.57 (−3.34 to 2.20)	9^f^
Retinal sensitivity (dB)^k^	14.1 (11.3 to 16.9)	13.7 (10.3 to 17.1)	−0.05 (−1.13 to 1.03)	6^e^	10.1 (5.79 to 14.4)	9.48 (4.99 to 14.0)	−0.62 (−1.39 to 0.15)	7^f^
Functional vision								
Reading speed (WPM)^l^^,m^	96.1 (14.9 to 177.3)	117.4 (31.7 to 203)	21.3 (−10.1 to 52.6)	28^n^	82.5 (20.6 to 144.4)	76.1 (9531.3 to 120.9)	−6.4 (−53.3 to 40.6)	7^f^
Quality of life								
LV-VFQ-48^o^								
Visual ability	1.70 (0.73 to 2.68)	1.87 (0.91 to 2.83)	0.16 (−0.42 to 0.75)	7^e^	1.51 (0.50 to 2.51)	1.92 (0.92 to 2.93)	0.42 (−0.05 to 0.78)	2^p^
Reading	1.27 (−0.31 to 2.86)	2.21 (0.44 to 3.97)	0.93 (−0.20 to 2.07)	2^h^	1.84 (0.51 to 3.17)	3.01 (0.76 to 5.26)	1.17 (0.06 to 2.28)	1^q^
Mobility	1.72 (0.92 to 2.52)	1.84 (1.13 to 2.55)	0.13 (−0.33 to 0.58)	6^e^	1.24 (0.28 to 2.21)	1.59 (0.57 to 2.62)	0.35 (−0.18 to 0.88)	4^f^
Visual information	2.00 (0.97 to 3.03)	1.75 (0.63 to 2.86)	−0.25 (−1.07 to 0.57)	5^e^	1.67 (0.44 to 2.90)	2.02 (1.06 to 2.97)	0.35 (−0.12 to 0.83)	3^f^
Visual motor	1.75 (0.90 to 2.60)	1.57 (0.69 to 2.44)	−0.19 (−0.74 to 0.37)	7^e^	1.40 (0.50 to 2.30)	1.64 (0.86 to 2.43)	0.24 (−0.32 to 0.80)	3^f^
COR^r^=2.2								
Total	8.45 (3.43 to 13.5)	9.23 (3.96 to 14.5)	0.79 (−2.48 to 4.06)	7^e^	7.66 (2.55 to 12.8)	10.2 (4.72 to 15.7)	2.54 (0.48 to 4.59)	W=2^p^

a BFT: biofeedback training.

b BFT: biofeedback training.

c LogMAR: logarithm of the minimum angle of resolution.

d COR =0.24.

e n=5; *P*>.2.

f n=6; *P*>.2.

g COR =0.11.

h n=5.1<*P*<.2.

i BCEA: bivariate contour ellipse area.

j COR =0.61.

k COR =1.6.

l WPM: words per minute.

m COR =8.6.

n n=8; *P*>.2.

o COR =0.44.

p n=6.05<*P*<.1.

q n=6.1<*P*<.2.

r COR: coeficient of repeatability.

Neither baseline group difference (ITT: Welsch *t* test: *t*=1.32, df=19.1, *P*=.19; PP: Welsch *t* test: *t*_19.5_=1.48, *P*=.17) nor postintervention changes (ITT: Welsch *t* test: *t*_18.5_=0.87, *P*=.37; PP: Welsch *t* test: *t*_16.1_=1.50, *P*=.16) reached significance in retinal sensitivity (Tables 4 and 5). Measures at 1- and 6-month post-intervention showed no changes between groups (Table S1 in Multimedia Appendix 1).

Contrast sensitivity was impaired in all participants, across all spatial frequencies at baseline, with no between-group differences (ITT: Welch *t* test, 2-tailed: *t*_12.0_=0.73, *P*=.48; PP: Welch *t* test: *t*_9_=1.52, *P*=.16). No differences were observed after intervention, nor at 1- and 6-month follow-up (Figure 1 in Multimedia Appendix 2).

Baseline reading speed heterogeneity was large: 2 participants were ≥200 wpm, three ranged 100‐200 wpm, and the remainder <100 wpm. Group means did not differ initially (ITT: Welch *t* test: *t*_10.6_=0.42, *P*=.68; PP: Welch *t* test: *t*_7.87_=0.26, *P*=.79). Data analysis showed no significant difference between BFT and BFT-VR groups in reading speed after intervention (ITT: Welch *t* test: *t*_10.5_=0.37, *P*=.48; PP: Welch *t* test: *t*_6.12_=0.84, *P*=.40). Pre- and postintervention analysis showed no statistical difference in the BFT group (ITT and PP analyses; Tables 4 and 5). However, the BFT-VR group showed a clinically significant increase in reading speed after the intervention, above COR (ITT:+13.2, 95% CI −10.8 to 37.3; PP:+21.3, 95% CI −10.1 to 52.6; COR=8.6 wpm). Measures at 1- and 6-month postintervention showed no changes between groups (Table S1 in Multimedia Appendix 1); however, a clinically significant decrease in reading speed was observed between postintervention (117.4, 95% CI 31.7 to 203) and 1-month follow-up (99.2, 95% CI 13.8 to 185; *Δ*=−18.2, 95% CI -50.7 to 14.3; COR=8.6).

Five domain scores for daily living activities, visual ability, reading, mobility, visual information, and visual motor, and a composite were analyzed. At baseline, no domain differed between arms (Table S1 in Multimedia Appendix 2). After training, both groups exhibited clinically meaningful gains in the reading subscale (BFT:+1.17, 95% CI 0.06 to 2.28; COR>0.44; Table 4) statistically significant for the BFT-VR group (ITT:+0.92, 95% CI 0.17 to 1.66; Wilcoxon ranked test: W=2, n=8, .02<*P*<.05; PP:+0.93, 95% CI −0.20 to 2.07; COR>0.44; Tables 4 and 5). No changes between groups were observed at 1- and 6-month follow-up (Table S1 in Multimedia Appendix 1).

### BFT-VR Group: Exploratory Outcomes

**Figure 3.**
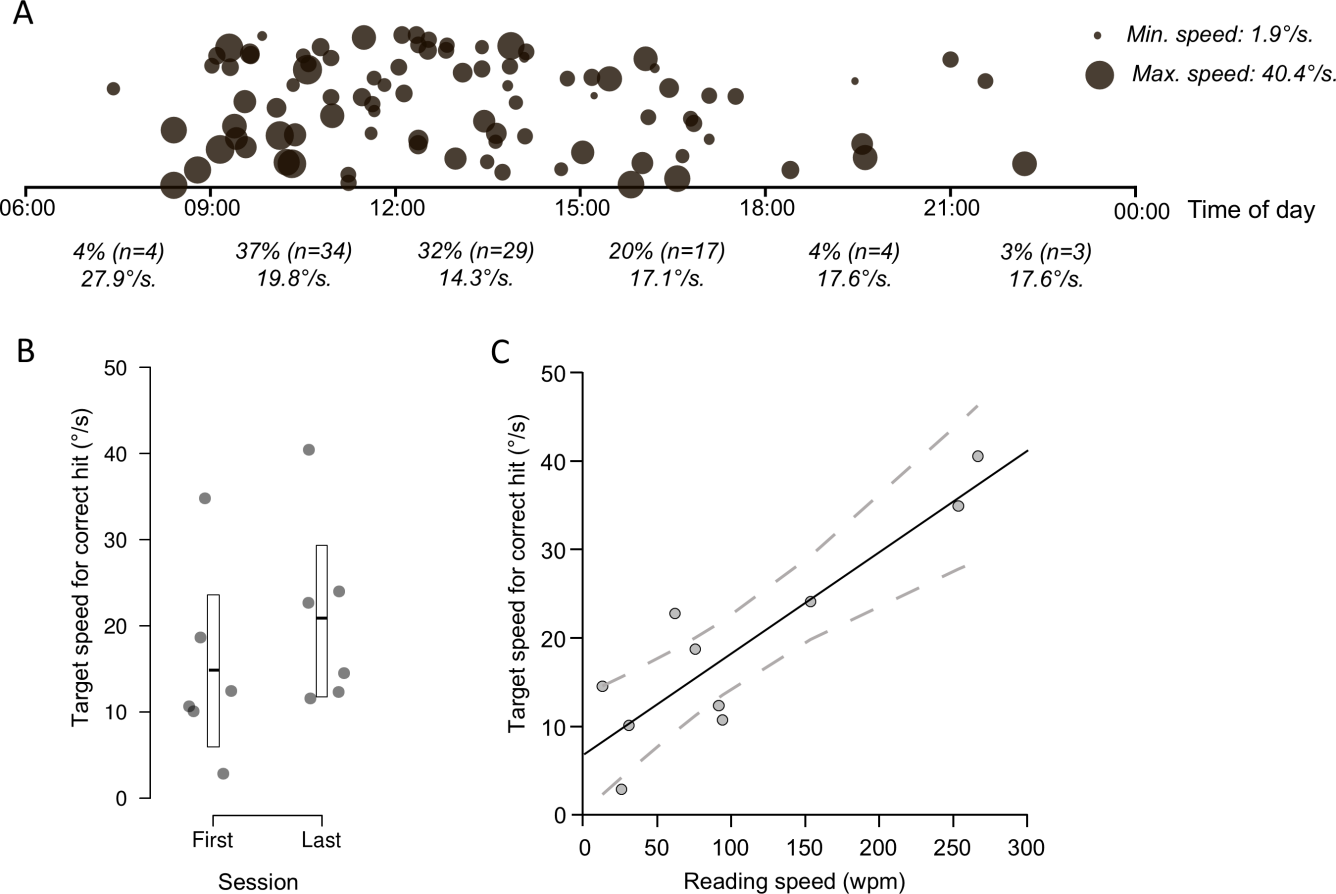
Exploratory outcomes. (A) Distribution of the session during the day. The size of the circle is correlated to the average speed of the target for a correct answer “ts” during the corresponding session. Percentage and number of sessions during the 3-hour bin are represented with the average speed of the target for a correct answer (=performance). (B) Performance (average speed of the target for a correct answer) at the 3D-SOT-VR (3D single object tracking in immersive virtual reality) stimulation for participants who completed the intervention at the first and last session (n=6). (C) Graph of the correlation between performance (the speed of the target for a correct answer in °/s) as a function of the reading speed (wpm). Pearson *R*=0.89; *P*<.001. Max: maximum; Min: minimum; wpm: words per minute.

The potential use of VR as a home-based interventional tool is very innovative; therefore, data about its use in real-world conditions is lacking. We recorded the time of day when participants who completed the intervention in the BFT-VR group performed the 3D-SOT-VR sessions. Session timestamps revealed that 74% (63/91) were performed between 9 AM and 3 PM (Figure 3A). Average total session duration—pre-VRISE, 3 × 5-min blocks, post-VRISE—was 28 (SD 7) minutes. ANOVA showed no interaction between time of day and tracking performance (*F*₅,₂=0.38; *P*=.83; Figure 3A).

Participants who completed the intervention (6/8) followed the same 3D-SOT-VR stimulation program for 4 weeks. Aggregate tracking performance increased: mean target speed on correct trials rose from 14.9°/second (95% CI 6.1 to 23.7) at the first session to 20.9°/second (95% CI 12.1 to 29.6) at the final session (Wilcoxon W=1, n=6, .05<*P*<.10; Figure 3B).

Reading speed was the only functional vision outcome that showed a clinically significant increase in the BFT-VR group, and participants showed variable tracking performance at the 3D-SOT-VR program. We therefore explored whether there is a correlation between the overall tracking performance at the 3D-SOT-VR stimulation and the reading speed. Reading speed correlated strongly with mean 3D-SOT performance (Pearson *r*=0.89; *P*<.001) across five participants with complete data (Figure 3C).

### Missing Data

Baseline data were complete for the five core clinical outcomes (far VA, near VA, retinal sensitivity, fixation stability, and reading speed). Missingness increased over follow-up, with 5/70 missing data at postintervention, 14/70 at 1-month follow-up, and 25/70 at 6-month follow-up. Little MCAR test on the baseline or postintervention core outcome matrix did not provide statistical evidence against the MCAR assumption (*χ*²_5_=5.8; *P*=.33). However, because of the very small sample size and documented reasons for missingness, including missed visits, withdrawal, and follow-up loss, this result was interpreted cautiously and not used as justification for multiple imputation.

## Discussion

### Principal Findings

This pilot feasibility study is the first to examine the combined use of clinical-based BFT and a tailored, home-delivered 3D-SOT-VR program for older adults with dry AMD. The main finding is one of partial feasibility rather than global feasibility. Adoption, safety monitoring, remote VR data transfer, and adherence among participants who continued the VR program were encouraging. In contrast, recruitment feasibility was not achieved, intervention completion in the BFT-VR arm did not reach the prespecified threshold, and follow-up attrition limited interpretation of longer-term outcomes. Recurrent suspensions under Ontario’s Emergency Management and Civil Protection Act between February 2021 and September 2022 hampered the recruitment rate, limited onsite testing capacity, and increased participants’ reluctance to travel and come to the hospital, fearing an increased risk of SARS-CoV-2 contamination. Altogether, this resulted in 10-fold slower enrollment than forecast. Similar setbacks have been reported in clinical trials embedded in health care systems during COVID-19 surges [46,47]. Post restriction, participants prioritized family gatherings and reconnecting with close relatives over the study’s requirements, leading to a loss of follow-up and increased no-shows. Nonetheless, the pandemic catalyzed broader public receptivity to digital and remote care solutions, even among older adults traditionally considered technophobic; our 75% completion rate underscores this shift. Previous studies, involving BFT and VR separately in older adults, have shown acceptable recruitment rates [7,10,48], although we cannot fully exclude that the combination of BFT and the audiovisual stimulation in VR might have increased the burden of the intervention.

Importantly, for participants enrolled during reopening periods and randomized in the BFT-VR group, a majority were able to complete the home-based 3D-SOT-VR intervention, in line with other studies indicating adoption of such technology in the older adult population [48,49]. A key benefit of home-based 3D-SOT-VR stimulation is its seamless integration into daily routines, as shown in other groups of participants [19], offering flexible and user-friendly use with reliable data transfer. Such benefits are even more important in the recent context of the pandemic, with the difficulty of travel, staff shortages, and related restrictions, as patients were unable to receive their treatment without delays. If VR potentially represents an adequate platform to deliver a visual rehabilitation program at home [18,19], it requires some adjustments tailored to the target population, especially older adults. Headset adjustments using straps and remote controllers seem to be troublesome for this population, hampering a full adoption or completion of the VR interventions.

Group-level VA, fixation stability, retinal sensitivity, contrast sensitivity, and quality-of-life estimates were imprecise and showed no consistent between-group benefit. Reading speed showed a clinically and statistically significant increase in the BFT-VR group, whereas no improvement was observed in the BFT, in contrast to previous studies [7,9,50,51]. Correspondingly, meaningful improvements were observed in patient-reported quality of vision questionnaires related to reading abilities in both groups. Individual reading-speed improvement in the BFT-VR group and its association with VR-task performance are potentially informative but should be interpreted as hypothesis-generating. A plausible mechanism is that dynamic target tracking may train visual attention, gaze allocation, head-eye coordination, and use of residual peripheral vision during eccentric viewing. However, the study did not directly measure PRL relocation stability, reading eye movements, cortical plasticity, or neural adaptation; therefore, the mechanism remains speculative. An important variable is the number of sessions of BFT delivered and total time of BFT. There is still no consensus on ideal exposure time for an optimal result [4], and previously we obtained significant improvement in visual functions and quality-of-life questionnaire using this scheme of four weekly sessions of 20 minutes (80 min in total) [7]. However, in a recent study that used two different intervals, 150 minutes in total delivered daily or on alternate days over 15 sessions, a statistically significant improvement was observed in VA, reading speed, fixation stability, and self-referred quality-of-life questionnaire in both groups [13]. The decrease in reading speed between postintervention and 1-month follow-up suggests that the short 4-week VR program may not be sufficient to induce sustained functional gains. This finding is compatible with clinical observations that patients may revert to less efficient viewing strategies after supervised rehabilitation if the trained strategy is not reinforced. It does not prove that indefinite treatment is required. Instead, future studies should test longer initial training, lower-burden maintenance sessions, or scheduled booster periods, while measuring whether PRL use and oculomotor behavior are stabilized over time.

The every-other-day schedule was chosen as a pragmatic compromise between repetition and tolerability. Weekly VR exposure was considered unlikely to add sufficient training beyond conventional weekly BFT, whereas daily exposure could increase fatigue, cybersickness risk, and nonadherence in older participants [48,49]. Future trials should include dose-finding components to compare training frequency, total exposure, and maintenance schedules. Such trials should also predefine whether the intended effect is short-term compensatory improvement, durable functional adaptation, or ongoing supportive therapy.

The ergonomics findings are clinically important. One participant discontinued the intervention because of headset weight and neck fatigue, and usability items related to headset adjustment and pointer selection were below threshold. This indicates that future implementation should prioritize lighter devices, improved weight distribution, simplified straps, larger and more accessible interfaces, gaze-based or controller-free selection, caregiver-assisted onboarding when needed, and remote troubleshooting. These design changes are particularly relevant for older adults with low vision, who may also have reduced manual dexterity, neck discomfort, or lower familiarity with immersive technologies [48,49].

### Limitations

The major limitation is the small sample size. Recruitment and study conduct were heavily disrupted by the COVID-19 pandemic and associated restrictions in Ontario, producing a marked reduction in recruitment compared with the prespecified criterion. As a consequence, the planned target of 32 participants was not reached, the precision of all secondary estimates was low, and between-group comparisons should not be interpreted as evidence for or against clinical effectiveness.

Additional limitations include open-label design, possible selection bias toward motivated participants who were willing to use VR at home, incomplete follow-up, and missing data that were not demonstrably MCAR. Because missingness was partly attributable to withdrawal, missed assessments, device burden, and follow-up loss, available-case analyses may be biased. Multiple imputation was not used because the sample size and sparse missing data structure would make imputation unstable and potentially misleading. The trial also did not include mechanistic measures of PRL use during reading, fixation strategy outside microperimetry, or neural adaptation, limiting interpretation of the reading-speed signal.

Taken together, these limitations mean that this study should be viewed as a formative feasibility trial. It identifies safety, remote monitoring, adherence among users, and preliminary functional signals that justify further study, but it also identifies recruitment, retention, missing data, long-term durability, and device ergonomics as barriers that must be solved before a definitive randomized trial.

### Conclusions

In conclusion, home-based VR visual stimulation combined with clinic-based BFT can be deployed safely in selected older adults with dry AMD and can generate objective remote adherence, safety, and performance data. The approach differs from conventional low-vision rehabilitation by moving part of the intervention into the home while retaining clinical supervision and remote monitoring. These results should not be interpreted as evidence of effectiveness; rather, they define the operational requirements for the next phase of research. Future trials should use optimized lightweight hardware, simplified interaction, larger multicenter recruitment, predefined missing-data handling, dose-finding of VR exposure, and mechanistic endpoints such as PRL stability, eye and head tracking, and reading-related oculomotor behavior.

## Supplementary material

10.2196/87596 Multimedia Appendix 1 1- and 6-month follow-up secondary outcome measures analyses (A. Intention-to-treat, B. Per Protocol).

10.2196/87596 Multimedia Appendix 2 Contrast sensitivity measures at 1- and 6-month follow-up in BFT and BFT-IVR groups.

10.2196/87596 Checklist 1 CONSORT e-HEALTH checklist.

## Abbreviations

3D-SOT-VR: 3D single object tracking in immersive virtual reality
AMD: age-related macular degeneration
BFT: biofeedback training
BFT-VR: biofeedback training in immersive virtual reality
CONSORT: Consolidated Standards of Reporting Trials
COR: coefficient of repeatability
dBHL: decibels hearing level
ICH: International Conference on Harmonisation
ITT: intention-to-treat
LogMAR: logarithm of the minimum angle of resolution
MCAR: Missing Completely At Random
PP: per protocol
PRL: preferred retinal locus
VA: visual acuity
VR: virtual reality
VRISE: Virtual Reality-Induced Symptoms and Effects
