# Supplementary material for: A Visual Telerehabilitation Program in Virtual Reality for Age-Related Macular Degeneration: Randomized Feasibility and Proof-of-Concept Trial
Source: JMIR Rehabil Assist Technol. 2026 Aug 3;13:e87596. doi: 10.2196/87596 (PMC13432248; doi:10.2196/87596)
Supplement: Multimedia Appendix 1 [file rehab-v13-e87596-s001.pdf]

Sup Table 1A 1- and 6-month follow-up analyses (ITT)

Function		Intervention (BFT-IVR)		Reference-controlled (BFT)	
		1M FU mean [95%CI]	6M FU mean [95%CI]	1M FU mean [95%CI]	6M FU mean [95%CI]
**Visual Function**					
Visual Acuity (near - LogMAR) *COR=0.24*		0.42 (95% CI 0.20 to 0.64)	0.35 (95% CI 0.23 to 0.48)	0.54 (95% CI 0.19 to 0.73)	0.46 (95% CI 0.19 to 0.73)
Visual Acuity (far - LogMAR) *COR=0.11*		0.59 (95% CI 0.48 to 0.82)	0.65 (95% CI 0.48 to 0.82)	0.59 (95% CI 0.30 to 0.88)	0.65 (95% CI 0.29 to 1.01)
Fixation Stability (BCEA 63%, $^{\circ 2}$) *COR=0.61*		2.26 (95% CI 1.06 to 3.47)	2.82 (95% CI 1.64 to 4.20)	3.73 (95% CI 0.53 to 6.93)	3.78 (95% CI 0.92 6.63)
Retinal Sensitivity (dB) *COR=1.6*		12.0 (95% CI 8.5 to 15.6)	14.6 (95% CI 11.8 to 17.4)	9.92 (95% CI 4.48 to 15.4)	11.6 (95% CI 6.07 to 17.2)
**Functional Vision**					
Reading Speed (wpm) *COR=8.6*		102.0 (95% CI 38.6 to 165.3)	93.0 (95% CI 38.1 to 147.9)	78.7 (95% CI 25.2 to 132.1)	76.3 (95% CI 23.0 to 129.6)
**Quality of Life**					
LV-VFQ-48 *COR=0.44*	Visual Ability	1.58 (95% CI 0.65 to 2.51)	2.21 (95% CI 1.40 to 3.02)	1.33 (95% CI -0.17 to 2.84)	1.88 (95% CI 0.42 to 3.33)
	Reading	1.85 (95% CI 0.28 to 3.42)	2.67 (95% CI 0.97 to 4.37)	2.91 (95% CI -0.40 to 6.22)	3.41 (95% CI 0.76 to 6.07)
	Mobility	1.57 (95% CI 0.81 to 2.34)	2.46 (95% CI 2.22 to 2.70)	0.73 (95% CI -0.68 to 2.13)	1.36 (95% CI -0.18 to 2.90)
	Visual Information	1.59 (95% CI 0.69 to 2.49)	2.11 (95% CI 1.00 to 3.23)	1.40 (95% CI -0.12 to 2.93)	1.94 (95% CI 0.18 to 3.69)
	Visual Motor	1.37 (95% CI 0.42 to 2.32)	1.91 (95% CI 1.27 to 2.56)	1.15 (95% CI -0.04 to 2.33)	1.50 (95% CI 0.34 to 2.66)
COR=2.2	Total	8.07 (95% CI 3.25 to 12.9)	11.37 (95% CI -0.40 to 8.92)	7.52 (95% CI -1.10 to 16.1)	10.09 (95% CI 2.19 to 18.0)

**Welsch test BFT vs BFT-IVR 1M FU**	**Welsch test BFT vs BFT-IVR 6M FU**
df=6.33, t=.65, p=.54	df=3.9, t=.47, p=.67
df7.29, t=.23, p=.81	df=3.35, t=.001, p=1.00

df=4.95, t=.83, p=.45	df=3.46, t=.33, p=.76
df=6.1, t=.71, p=.47	df=4.11, t=.58, p=.55
df=10, t=.55, p=.61	df=6.98, t=.39, p=.72
df=7.16, t=.96, p=.43	df=3.19, t=.90, p=.48
df=5.89, t=1.19, p=.23	df=3.67, t=.74, p=.55
df=6.55, t=1.42, p=.20	df=2.25, t=1.36, p=.31
df=6.68, t=.53, p=.61	df=3.99, t=.14, p=.90
df=6.13, t=.98, p=.36	df=3.96, t=.51, p=.64
df=6.78, t=.11, p=.92	df=3.93, t=.224, p=.83

Sup Table 1B 1- and 6-month follow-up analyses (PP)

Function		Intervention (BFT-IVR)		Reference-controlled (BFT)	
		1M FU mean [95%CI]	6M FU mean [95%CI]	1M FU mean [95%CI]	6M FU mean [95%CI]
**Visual Function**					
Visual Acuity (near - LogMAR) *COR=0.24*		0.32 (95% CI 0.14 to 0.50)	0.35 (95% CI 0.23 to 0.48)	0.54 (95% CI 0.19 to 0.73)	0.46 (95% CI 0.19 to 0.73)
Visual Acuity (far - LogMAR) *COR=0.11*		0.54 (95% CI 0.40 to 0.68)	0.65 (95% CI 0.48 to 0.82)	0.59 (95% CI 0.30 to 0.88)	0.65 (95% CI 0.29 to 1.01)
Fixation Stability (BCEA 63%, $°^2$) *COR=0.61*		2.04 (95% CI 0.87 to 3.21)	2.92 (95% CI 1.64 to 4.20)	3.73 (95% CI 0.53 to 6.93)	3.78 (95% CI 0.92 6.63)
Retinal Sensitivity (dB) *COR=1.6*		14.5 (95% CI 11.5 to 17.5)	14.6 (95% CI 11.8 to 17.4)	9.92 (95% CI 4.48 to 15.4)	11.6 (95% CI 6.07 to 17.2)
**Functional Vision**					
Reading Speed (wpm) *COR=8.6*		99.2 (95% CI 13.8 to 184.6)	93.0 (95% CI 28.0 to 158)	78.7 (95% CI 25.2 to 132.1)	76.3 (95% CI 23.0 to 129.6)
**Quality of Life**					
LV-VFQ-48 *COR=0.44*	Visual Ability	2.01 (95% CI 0.99 to 3.03)	2.21 (95% CI 0.89 to 3.53)	1.33 (95% CI -0.17 to 2.84)	1.88 (95% CI 0.42 to 3.33)
	Reading	2.34 (95% CI 0.32 to 4.36)	2.67 (95% CI -0.11 to 5.44)	2.91 (95% CI -0.40 to 6.22)	3.41 (95% CI 0.76 to 6.07)
	Mobility	2.18 (95% CI 1.43 to 2.93)	2.46 (95% CI 2.07 to 2.85)	0.73 (95% CI -0.68 to 2.13)	1.36 (95% CI -0.18 to 2.90)
	Visual Information	1.95 (95% CI 0.84 to 3.05)	2.11 (95% CI 0.29 to 3.93)	1.40 (95% CI -0.12 to 2.93)	1.94 (95% CI 0.18 to 3.69)
	Visual Motor	1.81 (95% CI 1.05 to 2.57)	1.91 (95% CI 0.86 to 2.97)	1.15 (95% CI -0.04 to 2.33)	1.50 (95% CI 0.34 to 2.66)
COR=2.2	Total	10.3 (95% CI 4.94 to 15.6)	11.4 (95% CI 4.48 to 18.2)	7.52 (95% CI -1.10 to 16.1)	10.1 (95% CI 2.19 to 18.0)

**Welsch test BFT vs BFT-IVR 1M FU**	**Welsch test BFT vs BFT-IVR 6M FU**
df=5.46, t=1.06, p=.33	df=3.9, t=.47, p=.67

df=5.58, t=.25, p=.82	df=3.35, t= .001, p=.99
df=5.33, t=.75, p=.49	df=3.46, t=.34, p=.76
df=5.92, t=1.00, p=.34	df=4.11, t=.58, p=.55
df=6.72, t=.40, p=.70	df=6.98, t=.39, p=.72
df=7.03, t=.73, p=.48	df=3.96, t=.34, p=.75
df=6.62, t=.29, p=.78	df=3.99, t=.38, p=.72
df=6.11, t=1.79, p=.12	df=2.25, t=1.36, p=.31
df=7.29, t=.57, p=.59	df=3.99, t=.14, p=.90
df=6.82, t=.92, p=.39	df=3.96, t=.51, p=.64
df=6.68, t=.53, p=.61	df=3.93, t=.24, p=.83
