# Supplementary figures and images for: A Visual Telerehabilitation Program in Virtual Reality for Age-Related Macular Degeneration: Randomized Feasibility and Proof-of-Concept Trial
Source: JMIR Rehabil Assist Technol. 2026 Aug 3;13:e87596. doi: 10.2196/87596 (PMC13432248; doi:10.2196/87596)

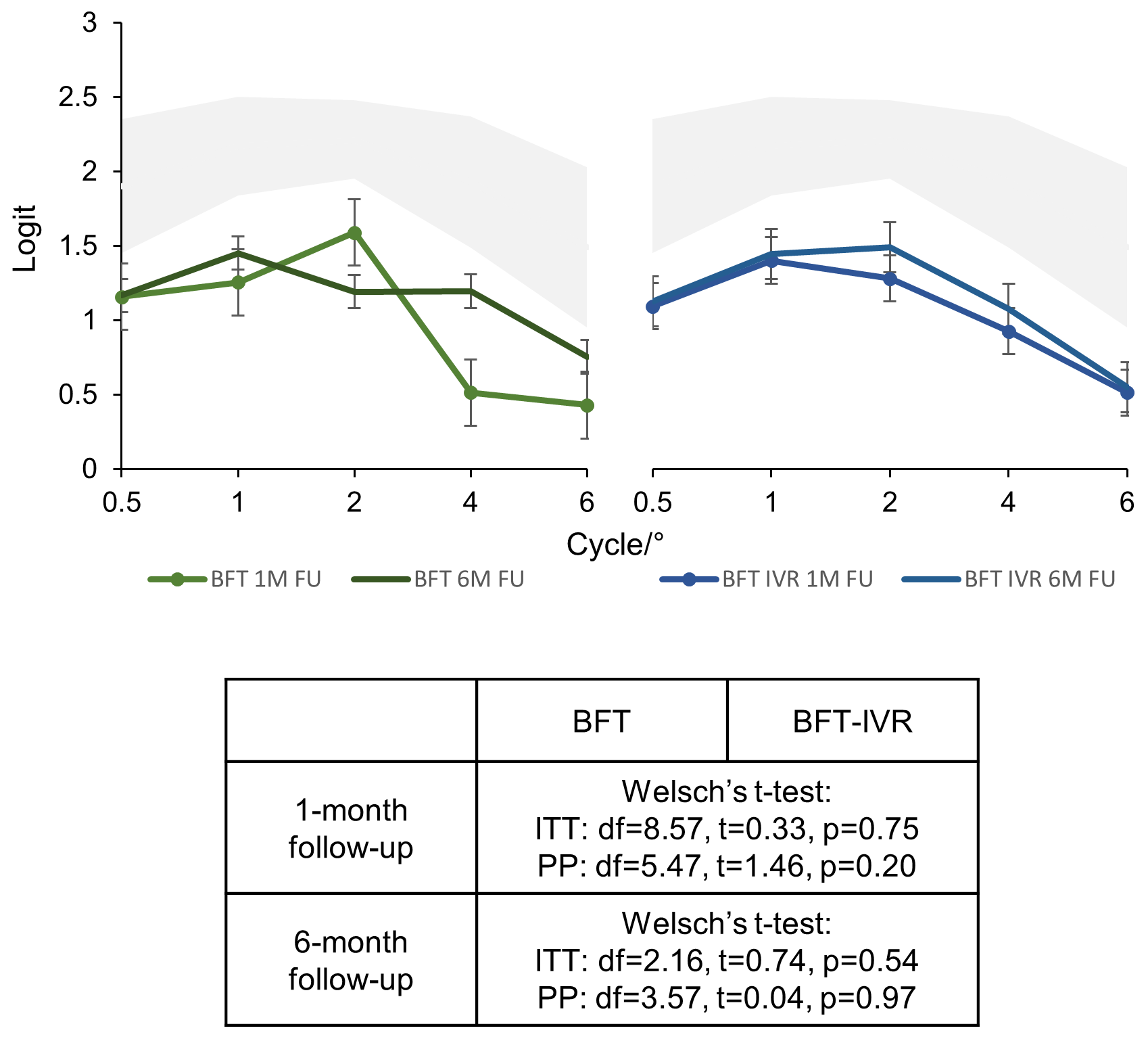

Supplement: Multimedia Appendix 2 [file rehab-v13-e87596-s002.png]
